# Structural Basis of Substrate Binding in WsaF, a Rhamnosyltransferase from *Geobacillus stearothermophilus*

**DOI:** 10.1016/j.jmb.2010.01.035

**Published:** 2010-03-26

**Authors:** Kerstin Steiner, Gregor Hagelueken, Paul Messner, Christina Schäffer, James H. Naismith

**Affiliations:** 1 Centre for Biomolecular Sciences, University of St. Andrews, North Haugh, St. Andrews, Fife KY16 9ST, UK; 2 Department of NanoBiotechnology, Vienna Institute of BioTechnology, Universität für Bodenkultur Wien, Muthgasse 11, A-1190 Wien, Austria

**Keywords:** SeMet, selenomethionine, SER, surface entropy reduction, ITC, isothermal titration calorimetry, crystal structure, *Geobacillus stearothermophilus*, rhamnosyltransferase, S-layer protein glycosylation

## Abstract

Carbohydrate polymers are medically and industrially important. The S-layer of many Gram-positive organisms comprises protein and carbohydrate polymers and forms an almost paracrystalline array on the cell surface. Not only is this array important for the bacteria but it has potential application in the manufacture of commercially important polysaccharides and glycoconjugates as well. The S-layer glycoprotein glycan from *Geobacillus stearothermophilus* NRS 2004/3a is mainly composed of repeating units of three rhamnose sugars linked by α-1,3-, α-1,2-, and β-1,2-linkages. The formation of the β-1,2-linkage is catalysed by the enzyme WsaF. The rational use of this system is hampered by the fact that WsaF and other enzymes in the pathway share very little homology to other enzymes. We report the structural and biochemical characterisation of WsaF, the first such rhamnosyltransferase to be characterised. Structural work was aided by the surface entropy reduction method. The enzyme has two domains, the N-terminal domain, which binds the acceptor (the growing rhamnan chain), and the C-terminal domain, which binds the substrate (dTDP-β-l-rhamnose). The structure of WsaF bound to dTDP and dTDP-β-l-rhamnose coupled to biochemical analysis identifies the residues that underlie catalysis and substrate recognition. We have constructed and tested by site-directed mutagenesis a model for acceptor recognition.

## Introduction

Glycosyltransferases play an important role in all living organisms. They are involved in the biosynthesis of glycoproteins, glycolipids, polysaccharides, and an array of secondary metabolites.^1^ Glycosyltransferases are grouped based on sequence similarity into currently 92 distinct families [CAZy (Carbohydrate-Active EnZYme) database†^2^ The classification of these families is based on experimentally characterised proteins.^3^

**Fig. 1:**
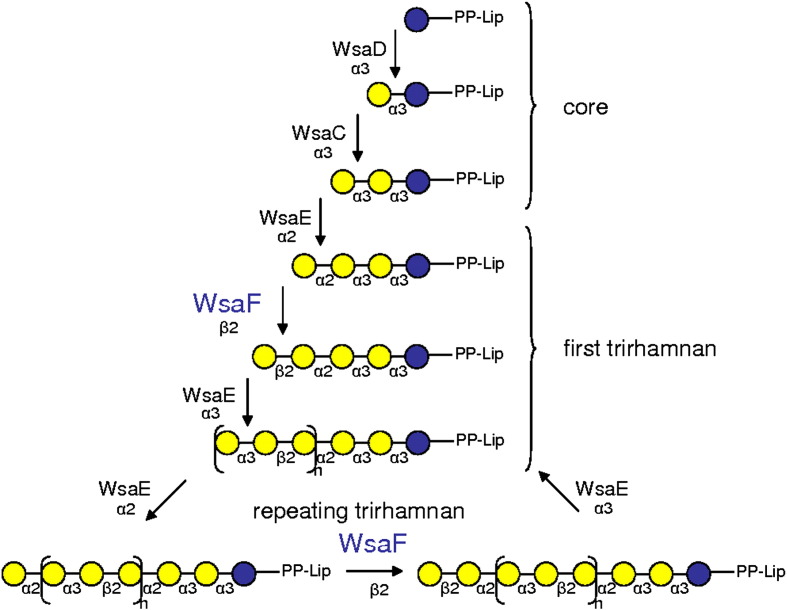
Action of the different rhamnosyltransferases in the polyrhamnan biosynthesis of the S-layer glycan in *G. stearothermophilus* NRS 2004/3a. Blue circle, galactose; yellow circle, rhamnose.^6^

*Geobacillus stearothermophilus* NRS 2004/3a serves as model organism for investigating the S-layer protein *O*-glycosylation pathway of Gram-positive bacteria.^4^ Recently, the function and substrate specificity of a lipid carrier transferase, different rhamnosyltransferases, and a methyltransferase involved in the S-layer protein polyrhamnan biosynthesis of *G. stearothermophilus* NRS 2004/3a have been elucidated.^5,6^ In the first step of the pathway, WsaP transfers a galactose residue from its nucleotide-activated form (UDP-Gal) to a membrane-associated lipid carrier at the cytoplasmic face of the plasma membrane.^5^ This is followed by a sequence of two α-1,3-rhamnosyltransferases WsaC and WsaD, which add the rhamnose (Rha) to build up the → 2)-α-l-Rha*p*-[(1 → 3)-α-l-Rha*p*-]_*n*__ = 1–2_ (1 → 3) linker (Fig. 1). Chain extension is accomplished by sequential addition of nucleotide-activated sugars by two processive rhamnosyltransferases WsaE and WsaF. This leads to an extended polysaccharide with a repeating trisaccharide motif [→ 2)-α-l-Rha*p*-(1 → 3)-β-l-Rha*p*-(1 → 2)-α-l-Rha*p*-(1 → ]_*n*__ = 13–18_. WsaE is a multifunctional enzyme forming both an α-1,2- and the α-1,3-linkage; WsaF is a β-1,2-rhamnosyltransferase (Fig. 1). Both enzymes utilise dTDP-β-l-rhamnose (dTDP-β-l-Rha) as the donor molecule. The terminal methylation of the glycan chain is catalysed by the methyltransferase domain of WsaE.^6^ The complete glycan chain would then be transported across the membrane by a process involving an ABC transporter and eventually transferred to the S-layer protein by the oligosaccharyltransferase WsaB.

Due to their ability to self-assemble into 2D crystalline nanolattices, S-layer proteins are very promising candidates for the design of tailor-made *neo*glycoproteins displaying the glycan in a highly ordered manner on the surface of bacteria or arrays.^7–9^ However, to be able to exploit the possibilities of engineering S-layer glycoproteins, it is important to understand the underlying biochemical mechanisms of S-layer glycan biosynthesis.

Here, we report the first structure of a rhamnosyltransferase. We have solved the structure of unliganded WsaF from *G. stearothermophilus* NRS 2004 and its complex with dTDP and dTDP-β-l-rhamnose, respectively. The complex structures and biochemical analysis of site-directed mutants identify the amino acid residues involved in substrate binding and those which are likely to play a role in the reaction mechanism.

## Results

### Sequence analysis of WsaF

In the CAZy database, WsaF is annotated to GT4 family, which is the largest retaining GT-B fold family with currently 11,446 entries. A BLAST search of the protein reveals proteins from more than 10 organisms with up to 46% identity, all of which are deposited as unknown proteins in the database derived from whole genome sequencing of bacteria (e.g., *Planctomyces maris*, *Acidovorax*, and *Magnetospirillum magnetobacterium*). However, all these bacteria also have genes that show homology to *rml* genes, which are involved in dTDP-β-l-Rha biosynthesis^10^ (the donor molecule for rhamnosyltransferases). A closer look reveals that ORF5 (open reading frame 5) from LPS biosynthesis gene cluster of *Xanthomonas oryzae* pv. Oryzae strain BXO8 (42% identity, E-value 2e^− 74^) is part of a cluster that has four other open reading frames that show strong sequence matches with wzm, wzt, WsaE, and WsaF of *G. stearothermophilus* NRS 2004/3a, indicating that these proteins also serve the same function in both organisms.^11^ The putative retaining GT4 glycosyltransferase WcrW (38% identity, E-value 2e^− ^^65^) is encoded by the CPS gene cluster of *Streptococcus pneumoniae* serotype 31, which displays CPS containing two β-linked rhamnoses, and we identify WcrW as a retaining rhamnosyltransferase.^12^ More distantly related are two proteins with assigned function, the retaining l-altrosyltransferase WbbX (25% identity, E-value 5e^− ^^7^)^13^ from *Yersinia enterocolitica* serotype O3 and the retaining fucosyltransferase WbsJ from *Geobacillus tepidamans* GS5-97^T^^4^ (Fig. S1).

### The overall structure of WsaF

**Fig. 2:**
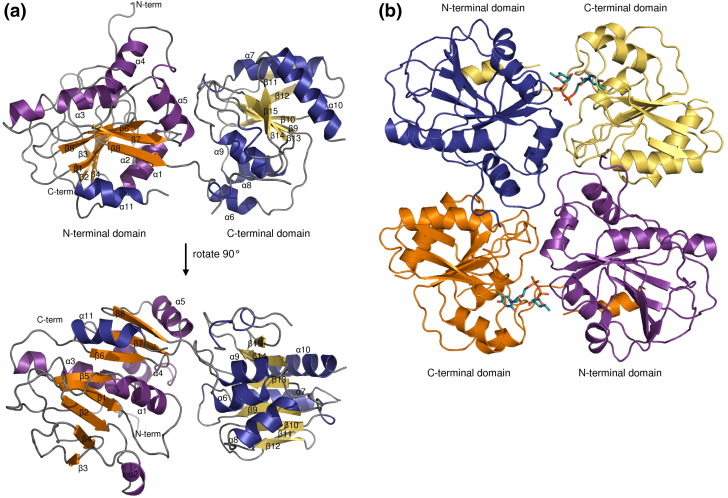
Structure of WsaF. (a) Cartoon presentation of a WsaF monomer. Helices are in purple and blue, strands are in orange and yellow, and coils are in grey. (b) Molecular dimer of WsaF in complex with dTDP-β-L-Rha (shown as stick model). The figures were depicted using PyMOL (DeLano Scientific).

The crystal structure of WsaF was determined using single-wavelength anomalous diffraction of selenomethionine (SeMet)-labeled WsaF crystals at 2.28 Å resolution (Table 1). The structure of WsaF consists of two domains with the typical GT-B-fold^14^ of two Rossmann-fold domains (β/α/β) and a cleft between the two domains, which includes the presumed catalytic centre (Fig. 2). The N-terminal domain comprises residues F26-F222, and the C-terminal domain is formed from residues T228-N381. The two domains are connected by the loop Q223-N227. The C-terminal α-helix S390-L413 crosses between the two domains and forms part of the N-terminal domain. The N-terminal domain has an eight-stranded β-sheet that is bounded by six α-helices. The C-terminal domain has a seven-stranded β-sheet that is flanked by five α-helices. Three regions of the structure have poor or no electron density, indicating conformational flexibility: the N-terminal M1-N25, the loop between β1 and α1 in the N-terminus (Q58-G63), and the loop that connects the C-terminal α-helix to the rest of the protein (N382-E389). In the crystal, two WsaF monomers form a dimer related by 2-fold symmetry. We examined the interface of this dimer using PISA (Protein Interfaces, Surfaces and Assemblies service at the European Bioinformatics Institute‡^15^). The dimer buries 1900 Å^2^ of solvent-accessible surface per monomer, and the statistical score provided by PISA indicates a dimer, which is also stable in solution. The interface is composed of residues from the N-terminal domain (α3, α4, loop α3/α4, and α5) of one chain and from the C-terminus (α7, loop β14/β15, loop β15/α10, and α10) of the other chain. The amino acid side chains involved mainly form hydrogen bonds and a few salt bridges. We tried to verify the existence of the dimer in solution with analytical gel filtration but the results were not definitive.

The DALI server^16^ identifies structural homologues belonging to the GT-B family. The closest structural match is the GT4 *N*-acetylglucosamine transferase MshA from *Corynebacterium glutamicum*, which is involved in mycothiol biosynthesis (DALI *Z*-score of 20.7, r.m.s.d. of 4.0 Å, and 11% sequence identity^17^) followed by a GT4 glycosyltransferase with unknown function, BaGT4 from *Bacillus anthracis* (*Z*-score of 20.3, r.m.s.d. of 4.7 Å, and 15% sequence identity^18^), the glycogen synthase from *Pyrococcus abyssi* (*Z*-score of 19.8, r.m.s.d. of 4.7 Å, and 12% sequence identity^19^), the GT4 α-1,3-glucosyltransferase WaaG from *Escherichia coli*, involved in lipopolysaccharide biosynthesis (*Z*-score of 19.0, r.m.s.d. of 3.9 Å, and 11% sequence identity^20^), and PimA, a GT4 phosphatidyl mannosyltransferase involved in phosphatidyl-*myo*-inositol mannoside biosynthesis of *Mycobacterium smegmatis* (*Z*-score of 17.5, r.m.s.d. of 4.6 Å, and 13% sequence identity^21^). Another GT4 family member involved in avilamycin A biosynthesis, AviT from *Streptomyces viridochromogenes* is more distantly related (*Z*-score of 13.0, r.m.s.d. of 5.4 Å, and 10% sequence identity^20^). Although the structural matches extend across both domains, the sequence conservation is only found in the C-terminal domain.

### WsaF_SER2_ structure

Due to difficulties in reproducing high-quality WsaF crystals, we engineered several mutants to improve crystallisability. In the surface entropy reduction (SER) approach, clusters of flexible, solvent-exposed, high-entropy amino acids, in particular K and E, are replaced by residues with lower conformational entropy, such as alanines.^22^ This may permit thermodynamically favourable crystal contacts. The WsaF mutants were expressed, purified, and screened with different crystallisation conditions, and the triple mutant WsaF_SER2_ (K78A/K79A/K81A) resulted in good-quality crystals. The mutated amino acids are located in the loop α1/β2, on the opposite site of the dimer interface and distant from the putative substrate and acceptor binding site. In the native crystals, only K78 (of the residues mutated) makes a crystal contact. The triple mutant is active and has an identical structure to native WsaF. All attempts to co-crystallise WsaF or WsaF_SER2_ with dTDP and dTDP-β-l-Rha failed, mainly resulting in precipitation of the protein. The structures of the dTDP-WsaF_SER2_ and dTDP-β-l-Rha-WsaF_SER2_ complexes were obtained by soaking and solved by molecular replacement using SeMet-WsaF as model. There are no major changes in the overall structure upon substrate binding.

### The structure of the dTDP-β-l-Rha (donor) binding site

**Fig. 3:**
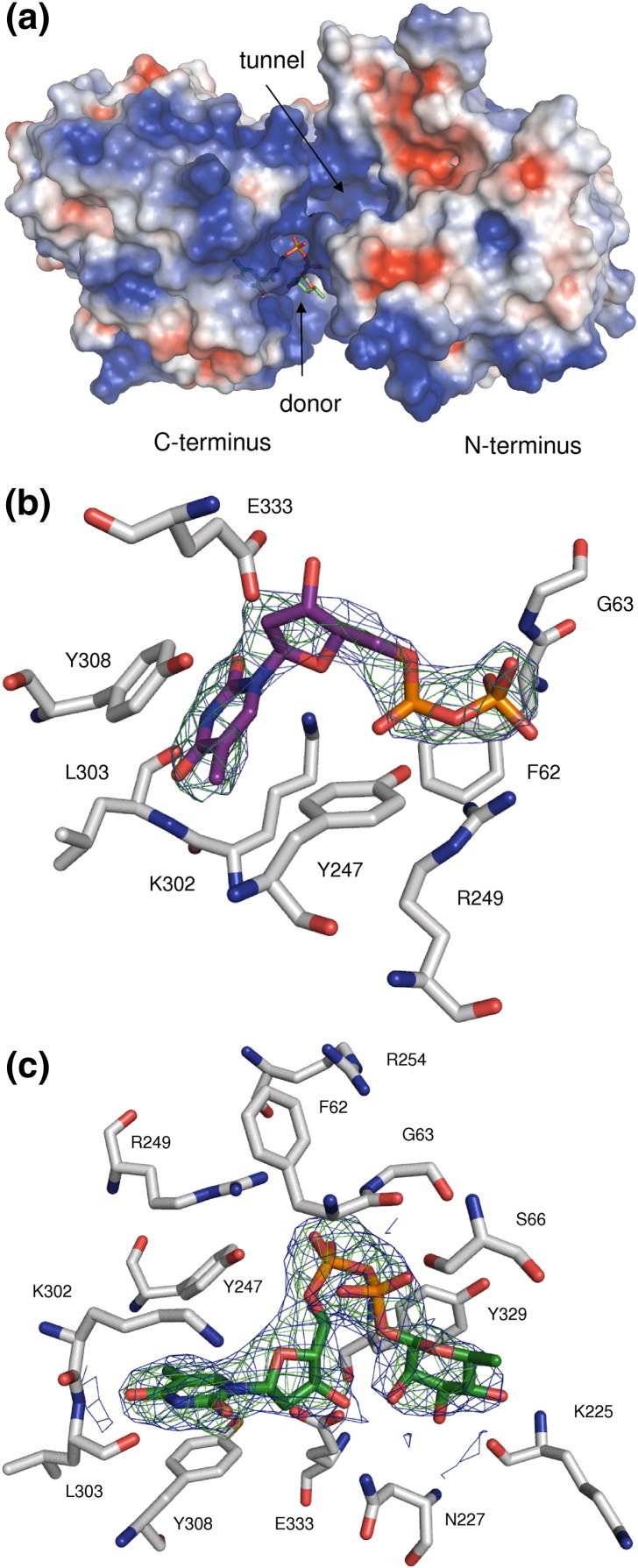
(a) Electrostatic surface of chain A of WsaF with dTDP-β-l-Rha bound in the binding pocket; electronegative regions are in red, electropositive regions are in blue, and neutral regions are in white (APBS plugin in PyMOL). (b) 2*F*_o_ − *F*_c_ (blue) and *F*_o_ − *F*_c_ (green) omit electron density map for dTDP. (c) 2*F*_o_ − *F*_c_ (blue) and *F*_o_ − *F*_c_ (green) omit electron density map for dTDP-β-l-Rha.

The substrate binding site is located in a cavity in the cleft between the two domains (Figs. 2b and 3a) and surrounded mainly by amino acid residues of the C-terminal domain. The structures with dTDP and dTDP-β-l-Rha, although overlapping the location of the thymidine ring and the ribose moieties, show significant differences for the diphosphate moieties. The majority of residues that interact with dTDP are located in the C-terminal domain, with the thymidine moiety forming polar interactions with the side chain of K302 and the main-chain atoms of L303 (with O2, N3, and O4 of thymidine) (Fig. 3b). The conformation of K302 is disordered in the native structure. Upon binding of dTDP (or dTDP-β-l-Rha), the side chain of K302 becomes ordered and the amine forms a hydrogen bond with O2 of thymidine. Y308 π stacks (distance 3.9–4.2 Å) with the pyrimidine ring of the dTDP, a common feature in nucleotide binding proteins.^23^ The CH_3_ group of the thymidine is involved in van der Waals interaction with V282 and G283. E333 hydrogen bonds with the O3 of the 2-deoxyribose. In dTDP-β-l-Rha, the pyrophosphate hydrogen bonds to the main-chain amide of G63 (Fig. 3c and Fig. S2a). R249 and K302 are approximately 4.5 Å from the pyrophosphate; a third positively charged residue, R254, is located about 7.5 Å from the pyrophosphate. F62 is located close to the pyrophosphate, but the side chain is disordered in all structures. The aromatic ring of Y329 makes a stacking interaction with the hydrophobic face of the rhamnose sugar; otherwise, the rhamnose residue only makes contacts with N227 (O2 with the side chain amide and O3 with the main-chain amide) and K225 (O3 and O4 with the main chain). In the dTDP complex, both phosphates make salt links (3.4 Å) with R249, the α-phosphate makes a salt link with K302 (4 Å) (Fig. 3b), and the β-phosphate makes a salt link with R254 (4.2 Å), which has a slightly different conformation as compared to the other structures. Overall, the loop between P325 and P329 changes the conformation slightly upon binding of dTDP. The side chain of H326 turns into the catalytic site. H326 is stabilised by a salt bridge to N201 (2.7 Å in native structure). The side chain of N201 shifts in the dTDP complex structure following H326. These conformational changes are not observed upon dTDP-Rha binding.

### The N-terminal domain of WsaF contains a tunnel that is suitable for acceptor binding

**Fig. 4:**
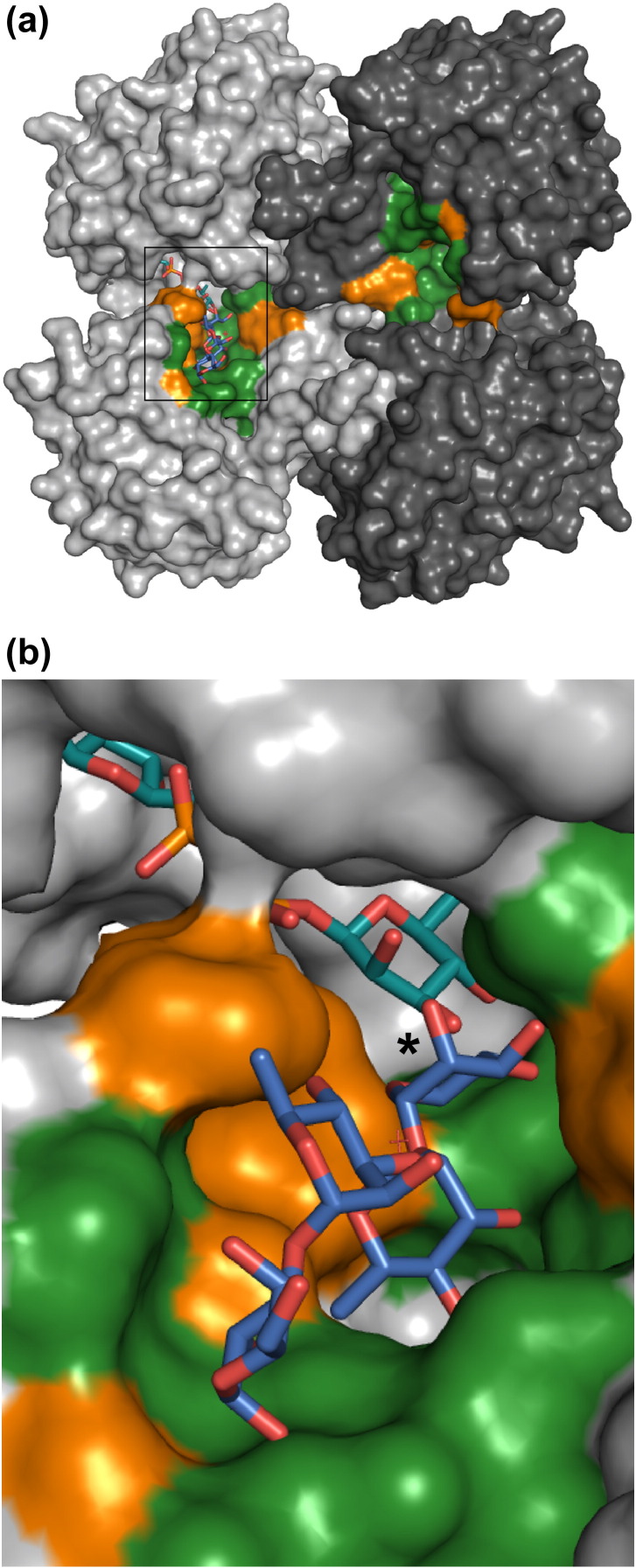
Molecular surface of WsaF with trirhamnosegalactose modelled into the putative acceptor binding tunnel of chain B. (a) Overview; (b) zoom into the tunnel region of chain B. dTDP-β-l-Rha, turquoise; acceptor, blue; chain B, light grey; chain A, dark grey; tunnel region, green; hydrophobic regions of the tunnel are depicted in orange.

The natural substrate for WsaF is the growing polyrhamnan chain linked via galactose and pyrophosphate to a lipid carrier.^6^ The shortest natural substrate would contain three rhamnose residues [α-l-Rha-(1–2)-α-l-Rha-(1–3)-α-l-Rha-(1–3)-d-Gal-(1,*O*)-PP-lipid] but would reach up to 20 repeating units (60 rhamnose residues) (Fig. 1). Our structural data suggest that as the C-terminal domain binds the donor, the N-terminal domain binds the acceptor. As even the simplest acceptor was not available in sufficient quantities for co-crystallisation experiments, we resorted to simple modelling based on the experimental structures. A tunnel mainly formed by amino acids of the N-terminus connects to the binding site of dTDP. The acceptor fragments α-l-Rha-(1–2)-α-l-Rha-(1–3)-α-l-Rha and α-l-Rha-(1–2)-α-l-Rha-(1–3)-α-l-Rha-(1–3)-α-d-Gal were positioned manually in the tunnel using PyMOL (Fig. 4). In this location, the side chain of R254 and the main chains of G64 and D88 would be predicted to form hydrogen bonds. Residues G63, I65, P54, S55, A140, Q170, D171, E173, and F176 would form van der Waals interactions. In this location, the acceptor replaces three water molecules in WsaF. Even for the shortest acceptor, the tetrasaccharide-PP-lipid, the pyrophosphate, and the lipid moieties would be located outside of the tunnel.

### Identification of important residues in the active site of WsaF by structure-guided mutagenesis

**Fig. 5:**
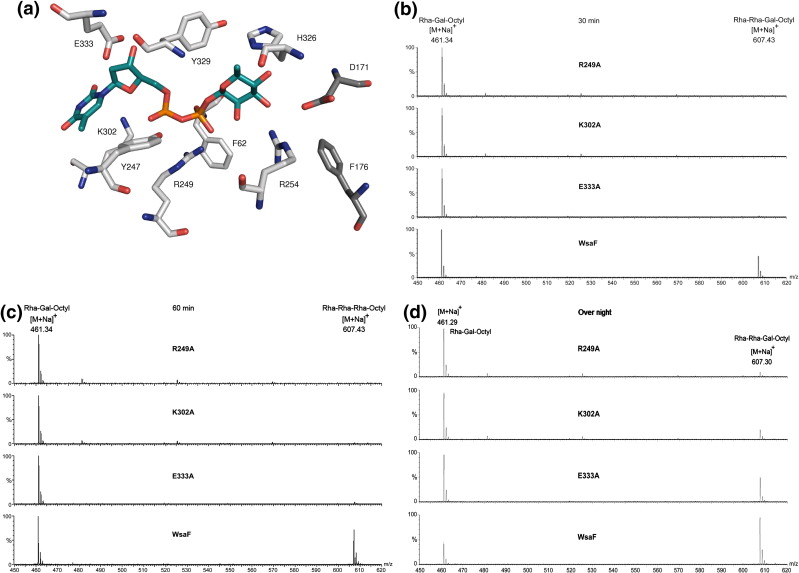
(a) Location of the mutated amino acids in the catalytic domain. (b–d) MS spectra of the singly charged ions at *m*/*z* 461.29 and 607.30 corresponding to the substrate and product of WsaF after 30 min, 60 min, and overnight reaction time.

The activity of WsaF was determined by incubating the enzyme with dTDP-β-l-rhamnose and the synthetic acceptor α-l-Rha-(1–3)-β-d-Gal-(1-*O*)-octyl.^6^ The conversion to the product was monitored at different time points by mass spectrometry. Activity was measured by calculating a ratio of the octyl acceptor and octyl products over time, and this was compared with native WsaF. The assay conditions were identical except for the protein to ensure reliability. However, we regard the assay data as qualitative (Table 2) rather than quantitative. WsaF is active over a wide pH range from 4 to 9. Only at the extremes does activity reduce due to precipitation. The location of the mutated residues is shown in Fig. 5a. Y247A was completely inactive, and D171A, F176A, R249A, R254A, and Y329A show only very weak activity after prolonged (overnight) incubation (Table 2 and Fig. 5b–d). Residues F62A and K302A show significantly reduced activity, with H326A and E333A showing less reduced activity (Table 2, and examples are given in Fig. 5b–d). CD measurement confirmed that all mutants were folded properly (data not shown). Isothermal titration calorimetry (ITC) of E333A and R254A showed weaker dTDP binding than native WsaF, when qualitatively comparing the curves (Fig. S3).

## Discussion

The low (15%) sequence identity between WsaF and structurally related enzymes makes sequence alignments essentially meaningless. Despite this, the structure shows that WsaF belongs to the GT4 superfamily of glycosyltransferases and is yet another example of how structure is conserved over sequence.

### The C-terminal domain of WsaF serves as donor-binding domain

The complex locates the donor binding site within the C-terminal domain. This is the first structure of a rhamnosyltransferase and allows the residues that control recognition and catalysis to be identified. Overall, the donor binding site is quite dissimilar to the other structures of the GT4 family, reflecting the profound chemical differences in the nature of the sugar donors. In both our complex structures, dTDP is placed at the interface between the domains. This position of the thymidine ring corresponds reasonably closely to the other nucleotide rings from other GT4 complexes.^17,20,21^ The failure of the phosphates to superimpose in the dTDP and dTDP-β-l-Rha complexes alerted us to the possibility of an artefact in the rhamnose position (caused by soaking in the substrate rather than co-crystallisation). Different locations of the sugar moiety were observed before, for example, in MurG^24^ and human UDP-galactose 4 epimerase.^25^ In both, the sugar moieties in the two subunits in the asymmetric unit have different conformations. Sugar nucleotides, which so strongly depend on the nucleotide for binding, could be particularly prone to producing such errors. The nucleotide would be bound in the correct location, but the sugar residue located in an alternative position. Glycosyltransferases can undergo significant conformational changes upon substrate and acceptor binding (e.g., MshA,^17^ PimA,^26^ and *E. coli* glycogen synthase^27^). In the GT4 UDP-GlcNAc transferase MshA, the binding of the donor to the C-terminus causes a 97° rotation of the N-terminus, enabling the formation of the acceptor binding site in close vicinity of the donor binding site. However, in other GT-B fold retaining enzymes, the conformational changes are less profound and usually caused by the motion of loops surrounding the active site.^28,29^ In WsaF, the loop Q58-G63, which is located close to the active site, shows poor electron density in all three structures, and the loop P325-Y329 shows some conformational changes upon dTDP binding. If significant conformational changes in WsaF are required to fully form the rhamnose pocket, then the soaking experiments, which, by their nature, work with protein already in the solid state, require care in their interpretation.

### Proposed mode of binding of dTDP-Rha to the C-terminal donor-binding domain

**Fig. 6:**
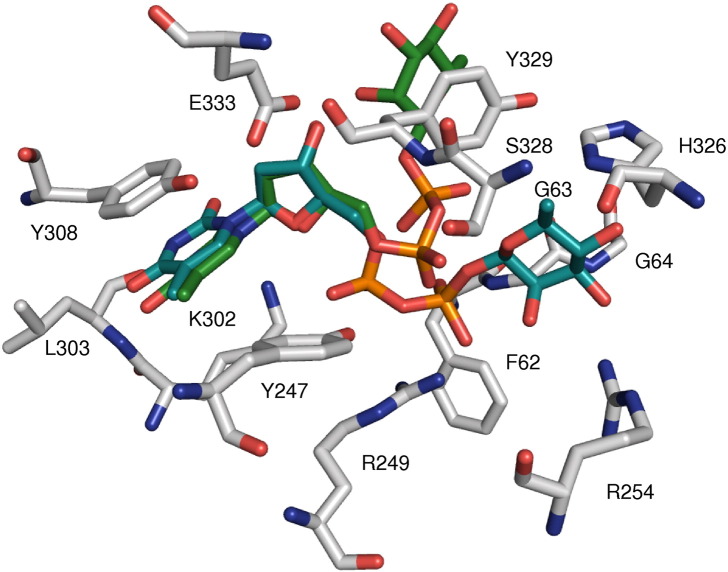
Overlay of the modelled dTDP-β-l-Rha (cyan) with dTDP-β-l-Rha (green) as seen in the structure in the catalytic domain.

The lack of sequence conservation at the active site between WsaF and other structurally characterised glycosyltransferases made the evaluation of the complex structure more difficult. In the end, we concluded that the location of the rhamnose ring is incorrect based on the following observations: its interactions with R249 and K302 are suboptimal, the ring makes few hydrogen bonds, the other glycosyltransferases locate the ring closer to the N-terminal domain, and, crucially, it is impossible to construct a plausible ternary complex in which the C1 of the ring could be attacked by any acceptor molecule. Of course, we cannot exclude a major conformational change that would allow the acceptor molecule to approach the rhamnose. However, we suggest that this is unlikely and have therefore constructed a revised position for rhamnose in dTDP-β-l-Rha by using the dTDP complex as a guide (Fig. 6). The dTDP shows interactions with R249, K302, and Y247, all of which are shown by mutagenesis to be important or critical for enzyme function. We suggest that these residues function by stabilising the pyrophosphate (leaving group), a role assigned for positively charged pairs in other members of GT4 family enzymes (PimA: R196 and K202, MshA: R231 and K236, WaaG: K209 and R208) and other GT-B retaining enzyme structures.^3,17,20,21^ R254 is placed immediately adjacent to the site of glycosidic bond formation where it could interact with both donor and substrate sugars as well as facilitate catalysis. Experimental data support an important role for R254, as R254A binds dTDP more weakly and is almost inactive. The modelled position of the rhamnose places it next to the disordered side chain of F62, which mutagenesis indicates is important but not critical for activity. Our model positions H326 close to the donor and acceptor sites, and the H326A mutant shows a reduced activity, consistent with recognition but not an essential catalytic role.

### The WsaF active site contains a possible signature sequence for rhamnose binding

E333 of WsaF binds to the O3 of 2-deoxyribose and corresponds with the second Glu of the EX_7_E motif found in the active site of all structures of GT4 family (PimA: E274 and E282, BaGT4: E282 and E290, AviT: E262 and E270, MshA: E316 and E324, WaaG: E281 and E289).^17,18,20,21^ The motif is widely conserved in retaining GT-B glycosyltransferases (family GT3, GT4, and GT20) and was first described for retaining α-mannosyltransferases.^30^ The mutant E333A showed a reduced binding affinity for dTDP and reduced enzyme activity consistent with a role in recognition but not in catalysis. In the other transferase structures, the first Glu interacts with the hydroxyl at position three of the donor sugar. In WsaF, the “would be” first Glu is in fact P325 and is remote from the donor. The nine-residue amino acid stretch starting at P325 has an unusual proline-rich motif, PHPSYPPLE, and the central tyrosine points towards the likely location of rhamnose. The proline richness gives this region of the active site a very different (and presumably constrained) orientation of the main chain compared to the other known GT4 structures (which have no proline residues in this region). The motif is conserved in all top BLAST hits, including the enzymes we predicted from sequence searches to be rhamnosyltransferases (Orf5^11^ and WcrW^12^) and may be a characteristic signature for rhamnose usage. We note that the two non-rhamnose glycosyltransferases that are related in sequence to WsaF have different motifs in this region. WsbJ,^4^ the fucosyltransferase (C3 and C5 epimer of rhamnose), has a TNLSLLPLE motif and WbbX,^13^ the altrose (6-deoxy-altrose is the C3 epimer of rhamnose)-utilising enzyme, has a TNLSYLPVE motif. As the central tyrosine is not absolutely conserved, it seems unlikely to be directly involved in catalysis; however, in WsaF, its mutation greatly reduces activity. We therefore suggest that the residue is involved in recognition of dTDP-β-l-rhamnose.

The N-terminal domain is likely to function as the acceptor binding region but unsurprisingly not conserved among the known structures of GT4 family due to the very different nature of the acceptors. Our model predicts that the acceptor binding site is located in a tunnel that is lined by D171 and F176. To support our model, we mutated both of these residues and confirmed that they profoundly impact on enzyme activity (Table 2).

### Catalytic mechanism of WsaF

The catalytic mechanism of retaining glycosyltransferases is still controversial.^3^ In the classic double-displacement retaining mechanism, a nucleophile attacks C1 from the β-face of the sugar residue, resulting in the formation of a glycosyl–enzyme intermediate.^31^ In the absence of an amino acid that could serve as nucleophile, an S_N_i-like reaction in which leaving-group departure and nucleophilic attack occur in a concerted but stepwise manner on the same face of the glycoside has been proposed (first reported in 1980 by Sinnott and Jencks^32^ for the solvolysis of glucose derivates). In the structure of WsaF, no potential nucleophilic amino acid is close to the sugar residue, suggesting that, similar to other GT4 family members, it too might follow the S_N_i reaction mechanism.^17,20,26^

## Conclusions

The large and expanding family of glycosyltransferases is an important topic for both therapeutic and biotransformation research. WsaF is the first rhamnosyltransferase to be structurally and biochemically characterised. The enzyme is an important player in the synthesis of the S-layer glycan, which has important applications in the manufacture of glycoconjugates.

## Materials and Methods

### Protein production and crystallisation

Recombinant native WsaF and SeMet variant WsaF from *G. stearothermophilus* NRS 2004/3a were expressed in *E. coli* BL21(DE3) Star, purified to homogeneity, and crystallised as described previously.^33^ The triple mutant WsaF_K78A/K79A/K81A_ (denoted WsaF_SER2_) was purified as the native protein. WsaF_SER2_ was crystallised by a hanging drop experiment [1 μL of protein solution (12 mg/mL) mixed with 1 μL of reservoir] using a reservoir of 0.2 mM Mg formate and 20% polyethylene glycol 3350. These crystals were soaked for 5 min in well solution containing 25 mM dTDP or 20 mM dTDP-β-l-Rha. The crystals were cryoprotected by addition of 20% glycerol to the well solution and flash frozen in liquid nitrogen. WsaF_SER2_, unlike native protein, gave crystals reproducibility.

### Structure determination and refinement

X-ray diffraction data from a single crystal of native WsaF were collected to a resolution of 3.0 Å on the ID14-4 beamline (λ = 0.979) at the European Synchrotron Radiation Facility (Grenoble, France) equipped with an ADSC Q315 detector. Since no suitable model for molecular replacement was available, we used a SeMet-labelled derivative of WsaF to solve the structure with experimental phases. Highly redundant X-ray diffraction data (redundancy of 14.4) from a single *P*2_1_ crystal of SeMet-labelled WsaF were collected to 2.28 Å resolution on the I03 beamline (λ = 0.9764) at Diamond (Didcot, UK) equipped with an ADSC Q315 detector. The data were processed and scaled with HKL2000.^34^ PHENIX.HYSS found 10 of the 16 SeMet residues in the asymmetric unit (two WsaF molecules). PHENIX.AUTOSOLVE was used to phase the data and to build an initial model of WsaF. The initial model was refined with PHENIX.REFINE^35^ and then manually improved in the molecular graphics program Coot.^36^ Subsequent refinement was carried out with REFMAC5 and TLS groups employed.^37^ Statistics are shown in Table 1. Difficulty in reproducing the crystallisation experiments prompted us to switch to WsaF_SER2_ for further structural studies. Data sets of the WsaF_SER2_-dTDP and WsaF_SER2_-dTDP-β-l-Rha complexes were collected in-house using Cu-Kα radiation on a Rigaku Saturn 944 detector (λ = 1.5418). The data were processed and scaled with XDS,^38^ and the structures were solved by molecular replacement using Phaser^39^ with the structure of the apoenzyme as search model. The dTDP and dTDP-β-l-Rha ligands were located in the initial difference Fourier maps and manually positioned using Coot. The ligands have higher *B*-factors than the protein, reflecting less than full occupancy. We felt that the resolution was insufficient to accurately model both *B*-factor and occupancy in the model; thus, occupancy was set to 1. Refinement was performed using REFMAC5^37^ and PHENIX.^35^

### Site-directed mutagenesis

Amino acids predicted to be involved in substrate recognition and reaction mechanism were mutated. In addition, to enhance the crystallisability of WsaF, we used the SER approach, and four candidates identified by the SER server§^22^ were generated including the triple mutant WsaF_SER2_. The following mutants WsaF_F62A_, WsaF_D171A_, WsaF_F176A_, WsaF_Y247A_ WsaF_R249A_, WsaF_R254A_, WsaF_K302A_, WsaF_H326A_, WsaF_Y329A_, and WsaF_E333A_ were generated for functional studies, and mutants WsaF_K240A/E240A/K242A_, WsaF_K78A/E79A/K81A_ (WsaF_SER2_), WsaF_E284A/K285A/K287A_, and WsaF_E108A/E109A/K111A_ were generated for crystallisation experiments. All mutants were made by following the QuikChange (Site-Directed Mutagenesis protocol developed by Stratagene (La Jolla, CA) but with the use of *Pfu* DNA Polymerase from Promega (Madison, WI) and DpnI from Fermentas (Burlington, Canada). The plasmid pET28a-WsaF was used as template.^6^ Mutations were confirmed by sequencing at the Dundee University sequencing unit. The proteins were expressed in *E. coli* BL21(DE3) and purified as described for wild-type WsaF.^33^ Protein integrity and identity were confirmed by mass spectrometry.

### Enzymatic assay

The biosynthesis of dTDP-β-l-rhamnose and the chemical synthesis of α-l-Rha-(1–3)-β-d-Gal-(1-*O*)-octyl were described previously.^6^ The assay reaction mixture contained 1 μL (20 nmol) of substrate, 1 μL (20 nmol) of dTDP-β-l-Rha, and 40 μg of purified WsaF or WsaF mutants in a final volume of 20 μL of 20 mM ammonium acetate, pH 8.0. Incubation was performed at 37 °C, and 2-μL samples were taken at different time points (30 min, 60 min, and 16 h). The samples were immediately flash frozen in liquid N_2_ and stored at − 80 °C until mass spectrometry analysis. The buffer was exchanged to 20 mM actetate (pH 4 and 5), 20 mM Na phosphate (pH 6, 7, and 8), and 20 mM Tris/HCl (pH 9) to evaluate the effect of pH range upon WsaF activity, and the assay was performed as described above.

For mass spectrometric analysis, the samples were diluted in 400 μL of 75% methanol containing 0.1% formic acid to a final concentration of 10 pmol/μL. Positive mode mass spectrometry was performed on a Micromass LCT ESI-ToF (Waters Micromass, Manchester, UK). Spectra acquisition was performed using 3 kV capillary and 40 V cone voltage. Desolvation gas flow was set at 600 L/h and that for cone gas was set at 30 L/h. Samples were injected at a flow rate of 20 μL/min, and spectra was acquired for 1 min. The instrument was controlled by MassLynx 4.0 software (Waters Micromass).

### Isothermal titration calorimetry

ITC was performed using a VP-ITC instrument (MicroCal Inc., Northampton, MA). The enzymes were dialysed against 20 mM NaP buffer, pH 8.5, containing 50 mM NaCl. The dialysis buffer was used to dissolve dTDP, and the pH was monitored. All solutions were degassed under vacuum prior to use. The calorimeter cell contained 1.4 mL of 20 μM WsaF or WsaF mutants, and the syringe contained 300 μL of 300 μM dTDP. Titrations were performed by a single preliminary injection of 2 μL of dTDP solution followed by 43 injections of 5 μL at 20 °C. The data were corrected for heats of dilution of dTDP. The raw data were integrated and fitted to a one-site model of binding using MicroCal Origin version 7.0.

### Accession numbers

Coordinates and structure factors have been deposited in the Protein Data Bank with accession numbers 2x0d (WsaF), 2x0e (WsaF-dTDP), and 2x0f (WsaF-dTDP-Rha).

## Figures and Tables

**Table 1:** Data collection and refinement statistics

	SeMet-labelled WsaF	WsaF_SER2_-dTDP	WsaF_SER2_-dTDP-Rha
*Data collection*			
Space group	*P*2_1_	*P*2_1_	*P*2_1_
Crystal system	Monoclinic	Monoclinic	Monoclinic
Unit-cell parameters			
*a*, *b*, *c* (Å)	75.9, 75.5, 78.1	75.8, 75.6, 77.7	75.9, 75.7, 77.5
α, γ, β (°)	90, 90, 108.3	90, 90, 103	90, 90, 102.8
Matthews coefficient (Å^3^ Da^− 1^)	2.18	2.17	2.16
Molecules per AU	2	2	2
Resolution range (Å)	47.3–2.28	29.6–2.81	28.8–2.55
Total observations	1,764,457	72,925	97,389
Unique reflections	37,051	38,750	52,047
Completeness (%)^a^	99.9 (98.9)	95.1 (68.3)	94.8 (66.9)
*R*_merge_ (%)^a^	15.8 (58.0)	8.1 (44.5)	8.2 (41.4)
*I*/σ(*I*)^a^	14.1 (3.3)	13.6 (2.6)	12.6 (2.5)
			
*Refinement statistics*			
Number of reflections used	37,017	38,728	52,027
*R*_free_ (%)	23.9	24.6	22.9
Number in test set for *R*_free_	1994	1971	2603
*R*_work_ (%)	19.3	18.9	18.6
Number of protein atoms	6259	6193	6241
Number of ligand atoms	—	54 (dTDP)	68 (dTDP-Rha)
Number of water molecules	191	118	212
r.m.s.d. bonds (Å)	0.009	0.006	0.005
r.m.s.d. angles (°)	1.2	1.0	1.0
Average *B*-factor (Å^2^)			
Protein	32.6^b^	34.9^c^	35.3^c^
Ligand	—	68.3^d^	55.4^d^
Water	31.4	26.2	32.7
Ramachandran statistics: favoured/outlier (%)	95.16/0.67	96.00/0.27	93.72/0.67

a Values in parentheses are those for the highest-resolution shell.

b Taken from REFMAC with TLS refinement.

c Total *B*-factor after refinement using PHENIX.

d The higher *B*-values for the ligands most likely result from an incomplete occupancy of the ligand due to the soaking experiment.

**Table 2:** Relative activity of the wild-type and mutant WsaF

Mutant	Activity	Location
Wild-type	^⁎⁎⁎⁎⁎^	
WsaF_SER2_	^⁎⁎⁎⁎⁎^	
F62A	^⁎⁎^	Acceptor
D171A	^⁎^	Acceptor
F176A	^⁎^	Acceptor
Y247A	—	Donor
R249A	^⁎^	Donor
R254A	^⁎^	Donor/acceptor
K302A	^⁎⁎^	Donor
H326A	^⁎⁎⁎^	Donor/acceptor
Y329A	^⁎^	Donor
E333A	^⁎⁎⁎^	Donor

^⁎⁎⁎⁎⁎^, activity of native protein, ^⁎⁎⁎^, significantly reduced activity, ~ 1/20; ^⁎⁎^, very reduced activity, ~ 1/40; ^⁎^, almost no activity.

## Footnotes

† http://www.cazy.org/

‡ http://www.ebi.ac.uk/msd-srv/prot_int/pistart.html

§ http://nihserver.mbi.ucla.edu/SER/
