# Supplementary material for: Structural Basis of Substrate Binding in WsaF, a Rhamnosyltransferase from Geobacillus stearothermophilus
Source: J Mol Biol. 2010 Mar 26;397(2):436–47. doi: 10.1016/j.jmb.2010.01.035 (PMC3898925; doi:10.1016/j.jmb.2010.01.035)
Supplement: Supplementary file 1 — Supporting material. [file mmc1.pdf]

SUPPORTING MATERIAL

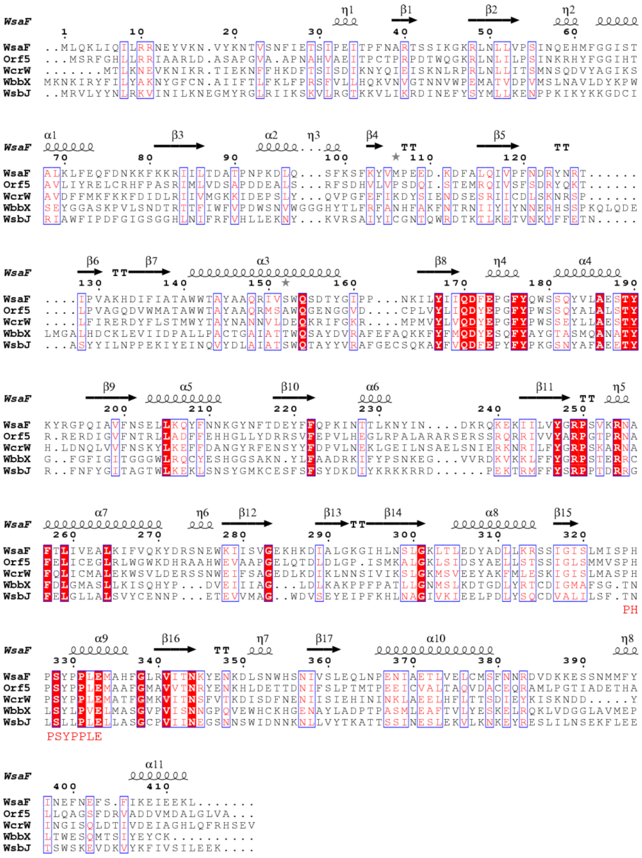

Fig. S1. Sequence alignment of WsaF (ESPript,[1]) with the sequence homologues ORF5 from LPS biosynthesis gene cluster of *Xanthomonas oryzae* pv. Oryzae strain BXO8, WcrW from *Streptococcus pneumoniae* serotype 31, WbbX from *Yersinia enterocolitica* serotype O3, and WbsJ from *Geobacillus tepidamans* GS5-97^{T}. Conserved residues are shown in a red background. The proline-rich sequence is written in red under the corresponding alignment.

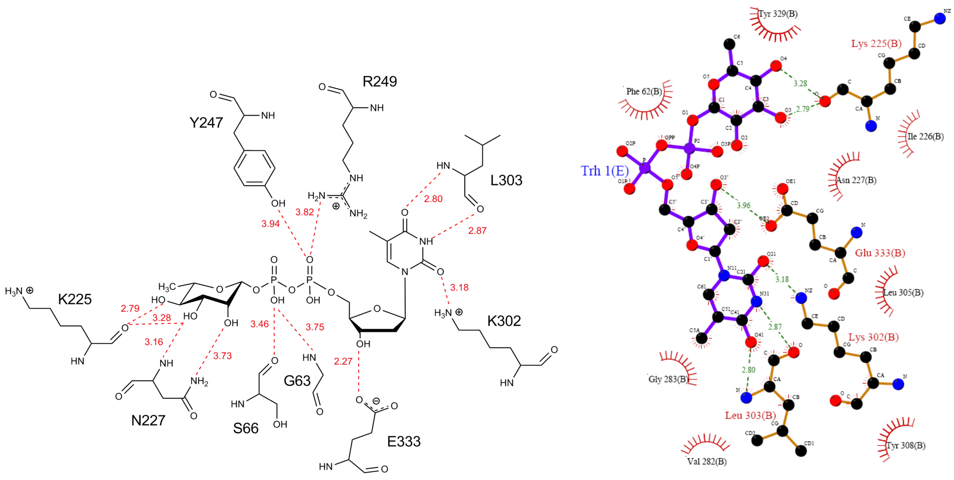

A B

Fig. S2. A: Schematic view of the dTDP-β-L-Rha protein interaction (ChemDraw). B: Diagram of WsaF interaction dTDP-β-L-Rha created using the program ligplot[2]. The residues that form hydrogen-bonds to dTDP-β-L-Rha are shown in ball-and-stick representation. Hydrogen bonds are presented as dashed lines and the interatomic distances are shown in Å. The residues that form van der Waals contacts with the dTDP-β-L-Rha are depicted as labeled arcs with radial spokes that point toward the ligand atoms with which they interact.

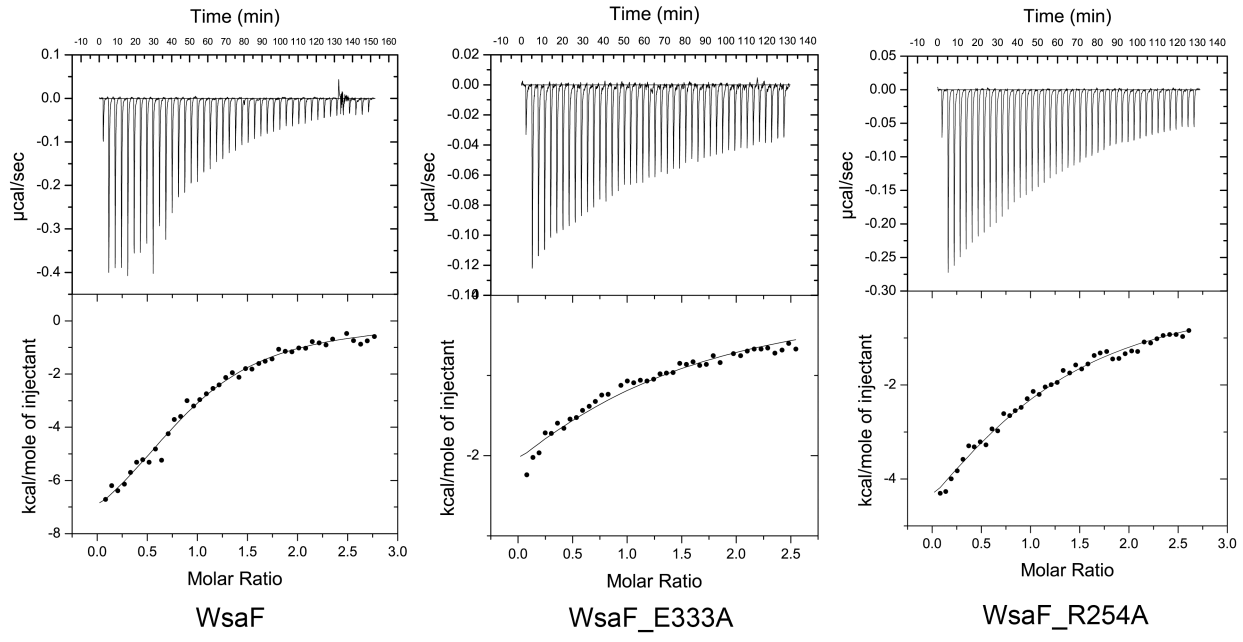

Fig. S3. ITC measurement of enzyme-ligand interactions. The upper panels show the raw data of the titration of WsaF or WsaFmutants with dTDP. The lower panels show the integrated heats of injections of the above titrations corrected for the heat of dilution and normalised to the ligand concentration. Solid lines correspond to the best fit of data using a one-site model of binding.

References

1. Wallace, A. C., Laskowski, R. A. & Thornton, J. M. (1995). LIGPLOT: a program to generate schematic diagrams of protein-ligand interactions. *Protein Eng.* **8**, 127-134.
2. Gouet, P., Courcelle, E., Stuart, D. I. & Metoz, F. (1999). ESPript: analysis of multiple sequence alignments in PostScript. *Bioinformatics* **15**, 305-308.
